# Structure–Activity Relationship Analysis of Flavonoids Isolated from the Leaves of *Erythroxylum rimosum* O. E. Schulz

**DOI:** 10.3390/molecules31101669

**Published:** 2026-05-15

**Authors:** Ivana P. dos Santos, Maria Eduarda V. Costa, Geovana A. de Oliveira, Francisnaira S. Santos, Débora O. S. Vitória, Robson Almeida Silva, Janaina M. C. do Vale, Gildeon S. Marques, Ademir Evangelista do Vale, Milena Botelho Pereira Soares, Taise de A. Araújo, André Braga Teles, Elisalva Teixeira Guimarães, Erika Maria de Oliveira Ribeiro

**Affiliations:** 1 Departamento de Ciências da Vida, Universidade Estadual da Bahia, Salvador 41150-000, Brazil; ivana.pfarma@gmail.com (I.P.d.S.); santosnaira89@gmail.com (F.S.S.); debivic02@gmail.com (D.O.S.V.); taiandradearaujo@gmail.com (T.d.A.A.); alteles@uneb.br (A.B.T.); etguimaraes@uneb.br (E.T.G.); 2 Escola Bahiana de Medicina e Saúde Pública, Salvador 41150-000, Brazil; mecosta2510@gmail.com (M.E.V.C.); geovana.alvesdeoli@gmail.com (G.A.d.O.); 3 Faculdade de Farmácia, Universidade Federal da Bahia, Salvador 40170-115, Brazil; robsonalmeida74@gmail.com (R.A.S.); janaynadovale@gmail.com (J.M.C.d.V.); gil_silv@hotmail.com (G.S.M.); advale@gmail.com (A.E.d.V.); 4 Instituto Gonçalo Moniz, Salvador 40296-710, Brazil; milena.soares@fiocruz.br; 5 Instituto SENAI de Inovação em Sistemas Avançados em Saúde (CIMATEC ISI SAS), Salvador 41650-010, Brazil

**Keywords:** 3-*O*-glycosylation, *Erythroxylum rimosum*, phytochemistry, antileishmanial, cytotoxicity

## Abstract

The flavonoids constitute a class of secondary metabolites distributed in plants, occurring in free form (aglycone) or conjugated sugars (glycosides), and are notable for their recognized pharmacological potential. This study investigated flavonoids isolated from the leaves of *Erythroxylum rimosum*, correlating their chemical structures with biological activities evaluated in vitro assays. The flavonoids did not show significant cytotoxicity in mammalian cells (CC_50_ > 100 μM), while flavonoids 4 and 5, glycosylated with six-carbon sugar, inhibited the promastigote forms of *L. braziliensis* with IC_50_ of 10.34 ± 0.70 μM and 10.14 ± 0.50 μM, respectively. In intracellular infection models, they reduced ~43% of parasitized cells and ~40% of amastigotes. Scanning electron microscopy demonstrated changes in promastigotes, including loss of membrane integrity and flagellum shortening. A molecular docking study with *L. mexicana* arginase suggested enzyme inhibition as a possible mechanism of action, influenced by sugar chirality. In the antioxidant evaluation, using the DPPH and *β*-carotene methods, quercetin showed the best inhibitory concentration value (IC_50_ = 0.22 ± 0.94 mg/mL), followed by glycoside 5 (IC_50_ = 0.42 ± 0.09 mg/mL). In addition, the flavonoids glycosylated with five-carbon sugar inhibited the acetylcholinesterase enzyme.

## 1. Introduction

The genus *Erythroxylum* has been recognized by the indigenous peoples of South America for its medicinal properties long before plant domestication, supported by modern scientific studies that reveal a rich variety of metabolites present in these species [1]. Molecular phylogeny research is unraveling the complexity and interactions within the genus, which underscores the need for further investigation to explore its vast medicinal potential [1]. Folk medicine already extensively uses species from this genus to treat various diseases, making it a promising source for discovering new bioactive properties. Although some species have already exhibited relevant biological activities, such as anesthetic, antioxidant, anti-inflammatory, cytotoxic, and anticancer effects, an in-depth study is still crucial to maximize the utilization of their medicinal resources [2].

Currently, the high cost and toxicity of synthetic drugs, coupled with limited access to healthcare services, have spurred the search for alternatives in plant-based medicines [3]. Plant-derived compounds are widely used as alternative medicine and are essential for promoting global health and well-being [4]. Many phytochemicals have become essential for the advancement of the pharmaceutical industry, increasing interest in researching their biological activities and potential as medicinal agents [5]. Evidence suggests that phytochemicals can lead to the development of new, more effective, and less toxic compounds with clinical applications in the treatment of various diseases [6].

Plants produce a wide range of organic compounds, categorized into primary and secondary metabolites. While primary metabolites are vital for plant development (performing structural and energy storage functions), specialized metabolites are crucial for survival and adaptation in adverse environments [7]. This latter category harbors the vast phytochemical arsenal with therapeutic potential. Among the various classes of specialized metabolites, flavonoids stand out as one of the most extensively investigated and promising groups. Structurally, they are polyphenolic compounds widely distributed in the plant kingdom, known for their broad spectrum of biological activities.

Fundamental reviews in the field have elucidated, for example, their potent antioxidant capacity, acting directly in the neutralization of reactive oxygen species (ROS) and in the chelation of metal ions, central processes in the prevention of chronic diseases associated with oxidative stress [8,9]. Furthermore, their notable anti-inflammatory activity is well-documented, involving the modulation of key cellular signaling pathways (such as the NF-κB pathway) and the inhibition of pro-inflammatory enzymes, including COX and LOX [10,11]. Other relevant biological activities, such as antitumor, antiviral, antimicrobial, and neuroprotective potential, have also been extensively reviewed, consolidating flavonoids as central targets in new drug discovery [12,13,14].

In this context, the *Erythroxylum* genus emerges as a notable source of phytochemicals, historically recognized for its diverse chemical profile, which includes not only alkaloids but also a rich variety of flavonoids, such as aglycones (quercetin, kaempferol) and their glycosylated derivatives [15,16]. Previous studies on compounds isolated from this genus have already reported significant biological activities [17,18]. To accelerate screening and deepen the mechanistic understanding of the potential of these compounds, in silico approaches have become strategic tools. Molecular docking, in particular, allows for the prediction of the binding mode and affinity of a ligand (flavonoid) to a specific molecular target (e.g., a key enzyme in a pathogen’s metabolism or a protein involved in inflammatory processes), providing a molecular rationale for the observed activity [19,20]. However, the assessment of potential biological activities of flavonoids isolated from *Erythroxylum* species, using an integrated approach that combines phytochemical characterization with experimental validation and computational prediction, still represents an underexplored field, warranting the investigation of new molecules from this genus.

Among these, flavonoids are abundant in aglycone (free) and glycosylated (bound to sugars) forms. Characterized by a 2-phenyl-chromone backbone, they are biosynthesized from acetic acid and phenylalanine derivatives via the shikimic acid pathway. Their classification is based on the degree of oxidation, the configuration of the C-ring, and the position of the B-ring attachment, which aids in understanding their properties and biological functions [21]. The glycosylated form of flavonoids enhances their solubility, stability in water, and bioavailability, in addition to potentially reducing toxicity and side effects [22,23]. Research on glycosylated flavonoids has been gaining relevance due to their numerous health benefits and therapeutic applications [24].

Their fundamental chemical structure significantly influences the bioactivity of flavonoids, the arrangement of the aromatic rings, the degree of oxidation, and the substitution pattern on the molecule [25]. The presence and location of substituents, such as hydroxyls and glycosides, can modulate their interactions with biological systems, their free radical scavenging capacity, their modulation of cellular signaling pathways, and their interaction with specific enzymes, conferring upon them multiple pharmacological targets and a vast therapeutic potential [26,27,28].

Therefore, the main objective of this study was to investigate the biological potential of isolated flavonoids. We aimed to highlight their ability to inhibit *L. braziliensis* promastigotes in vitro without significant toxicity to mammalian cells, their inhibitory activity against the acetylcholinesterase enzyme, and their notable antioxidant power.

## 2. Results

### 2.1. Isolated and Characterized Flavonoids

**Figure 1:**
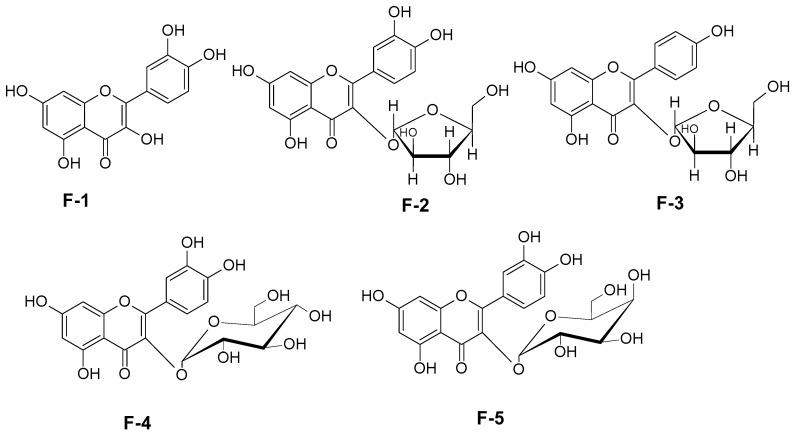
Quercetin (**F-1**), Kaempferol-3-*O*-*α*-arabinofuranoside (**F-2**), Quercetin-3-*O*-*α*-arabinofuranoside (**F-3**), Quercetin-3-*O*-*β*-glucopyranoside (**F-4**) and Quercetin-3-*O*-*β*-galactopyranoside (**F-5**). Source: Ribeiro et al. [28].

The chemical structures of the five flavonoids, isolated from the leaves of *Erythroxylum rimosum*, were previously elucidated by Ribeiro et al. [28] and are presented in Figure 1.

### 2.2. Absence of Cytotoxicity in Mammalian Cells

We investigated the cytotoxicity of the flavonoids in mammalian cells to evaluate the safety and potential application of these compounds. Flavonoids **3**, **4**, and **5**, which are structurally composed of quercetin linked to different sugars, did not demonstrate cytotoxic activity after 72 h of treatment, exhibiting a CC_50_ > 100 μM. In contrast, flavonoid **2**, structurally a derivative of kaempferol (which differs from quercetin by the absence of a hydroxyl group at the 3′ position of the B-ring and is linked to a pentose), presented a lower CC_50_ of 29.40 ± 0.30 μM. Gentian violet, used as the control, exhibited a CC_50_ of 0.30 ± 0.20 μM, making it approximately 98 times more cytotoxic than flavonoid **2**. This substantial difference reinforces the more favorable cytotoxicity profile of flavonoid **2** compared to a widely known (gentian violet) reference compound (Table 1).

### 2.3. Antileishmanial Potential of Glycosylated Flavonoids ***4*** and ***5***

Our in vitro assays revealed the promising potential of flavonoids **4** and **5** against *L. braziliensis* promastigotes. These glycosides presented IC_50_ values of 10.34 ± 0.70 μM and 10.14 ± 0.50 μM, respectively, while amphotericin B, the reference drug used for treating the disease, exhibited an IC_50_ of 2.28 ± 0.20 μM. Although amphotericin B demonstrated greater potency in vitro, the significant activity of flavonoids **4** and **5** positions them as important candidates for future investigations, especially considering natural compounds’ safety profile and cost-effectiveness (Table 1).

### 2.4. Selectivity Index and Therapeutic Potential

The Selectivity Index (SI), calculated as the ratio of cytotoxicity to macrophages (CC_50_) to the anti-parasitic activity (IC_50_), was found to be considerably higher for flavonoids **4** and **5** compared to amphotericin B. The SI values for compounds **4** and **5** were 9.6 and 9.8, respectively, demonstrating substantially superior selectivity when compared to amphotericin B, the first-choice drug for leishmaniasis treatment, which exhibited an SI of only 1.4 (Table 1). These results indicate that flavonoids **4** and **5** possess a notably lower toxicity towards J774 macrophages, concomitant with a high specificity in eliminating *L. braziliensis*.

### 2.5. Flavonoids ***4*** and ***5*** Reduce Intracellular Infection by L. braziliensis in Macrophages

**Figure 2:**
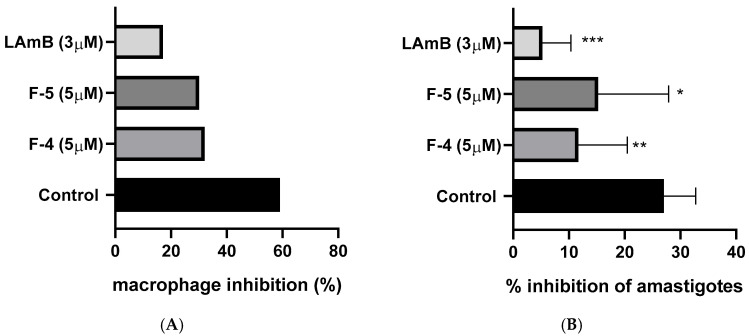
Effect of **F-4** and **F-5** against intracellular *Leishmania braziliensis* parasites. J774 macrophages were infected with *L. braziliensis* promastigotes and treated with **F-4** and **F-5** (5 µM) or amphotericin B (3 µM). After 24 h of treatment, the percentage of infected cells (**A**) and the mean number of amastigotes per field (**B**) were measured in 100 macrophages. * *p* < 0.05 vs. control (CTL), ** *p* < 0.01 vs. CTL-, *** *p* < 0.001 vs. CTL.

A significant reduction in the percentage of infected macrophages and the mean number of amastigotes per field was observed after 24 h of treatment (Figure 2). In the control group, approximately 70% of the cells remained infected. In contrast, treatment with flavonoid **5** (**F5**) at a concentration of 5 μM decreased the percentage of infected macrophages to about 45%, corresponding to a ~36% inhibition compared to the control. Flavonoid **4** (**F4**) was even more effective at the same concentration, reducing the infection to approximately 40% of macrophages, representing a ~43% inhibition. When evaluating the mean number of amastigotes per field, this efficacy pattern was maintained: **F5** reduced the number from ~30 to ~22 amastigotes per field (~27% inhibition), while **F4** achieved a decrease from ~30 to ~18 amastigotes per field (~40% inhibition).

Amphotericin B (3 μM), used as a positive control, demonstrated high efficacy, reducing the percentage of infected J774 macrophages from ~70% (control) to ~20% (~71% inhibition), and decreasing the mean number of amastigotes from ~30 to ~5 per field (an 83% inhibition).

In a direct comparison, **F4** (5 μM) inhibited ~43% of infected macrophages and ~40% of amastigotes, whereas **F5** (5 μM) showed inhibitions of ~36% and ~27%, respectively (Figure 2).

### 2.6. Morphological Changes in L. braziliensis Induced by Flavonoids ***4*** and ***5***

**Figure 3:**
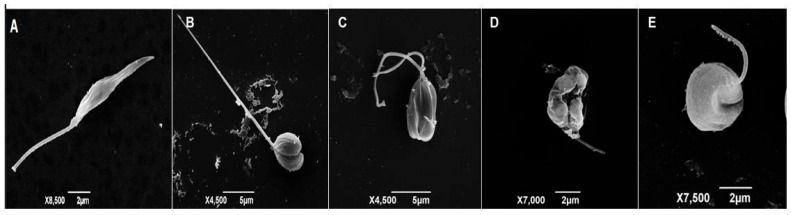
Untreated control (**A**) presents typical parasite morphology with an elongated shape, fusiform body and well-defined flagellum. Ultrastructural changes in *L. braziliensis* promastigotes after treatment with Quercetin-3-*O-β*-glucopyranoside (**B**,**C**), Quercetin-3-*O*-*β*-galactopyranoside (**D**,**E**) and untreated control (**A**) by scanning electron microscopy JSM-6390LV (JEOL).

We conducted scanning electron microscopy analysis to assess the effects of flavonoids **4** (Quercetin-3-*O*-*β*-glucopyranoside) and **5** (Quercetin-3-*O*-*β*-galactopyranoside) on the morphology of *L. braziliensis*. The results showed that these flavonoids induced significant morphological alterations in treated parasites when compared to the untreated control (Figure 3A). Among the observed changes were: loss of membrane integrity, formation of blebs, a shortened flagellum, and rounding and retraction of the cell body (Figure 3B–E). These findings provide visual evidence of the activity of flavonoids **4** and **5** (against the parasite’s structure, suggesting a mechanism of action that directly compromises its cellular integrity.

### 2.7. In Silico Interaction of Flavonoids ***4*** and ***5*** with Leishmania Arginase: Influence of Chirality and Recognition at the Catalytic Site

**Figure 4:**
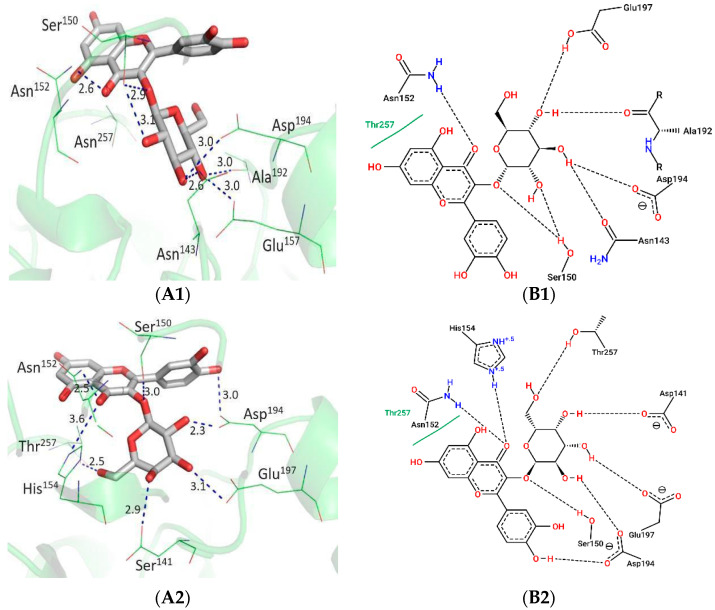
In silico analysis of the interaction between Quercetin-3-*O-β*-galactopyranoside (**A**) and Quercetin-3-*O-β*-glucopyranoside (**B**) with the catalytic site of *L. mexicana* arginase. In which: (**A1**) best docking pose of Quercetin-3-*O-β*-galactopyranoside, as classified by GOLD 5.3.0 software (**A2**) 2D representation of the amino acid residues involved in the interactions with Quercetin-3-*O*-*β*-galactopyranoside. (**B1**) Best docking pose of Quercetin-3-*O-β*-glucopyranoside according to GOLD 5.3.0. (**B2**) 2D scheme of the interactions between arginase residues and Quercetin-3-*O-β*-glucopyranoside. The binding site residues are represented in wire mode and the compounds in stick mode. Hydrogen bonds are indicated by dashed lines, with distances expressed in angstroms (Å). The three-dimensional Figures (**A1**,**B1**) were generated with PyMOL 1.3 software, and the two-dimensional representations (**A2**,**B2**) were produced with PoseView.

The activity of flavonoids against *Leishmania* arginases was investigated by molecular docking, using the LmARG structure (PDB = 4IU1) due to its high sequence identity with LbARG [29] (Supplementary Materials). Figure 4 illustrates the spatial orientations and interactions between the compounds and the residues of the LmARG catalytic site. The quercetin core of both flavonoids (**4** and **5**) exhibited similar spatial orientations and interactions with LmARG, acting as hydrogen bond acceptors from Ser150 and Asn152, and making hydrophobic interactions with Thr257. Only the catechol moiety of the quercetin core of flavonoid **4** came close enough to Asp194 (3.0 Å) to act as a hydrogen bond donor.

The chirality at the 4-carbon of the glucose and galactose moieties of flavonoids **4** and **5**, respectively, determined different types of interactions with the arginase: the amino acids Ala192, Asn143, and Ser150 were accessed only by the glucose of flavonoid **4**; residues Asp141 and Thr257 interacted only with the galactose of flavonoid **5**; and the amino acids Asp194 and Glu197 established interactions with both the glucose and the galactose.

### 2.8. Flavonoids Act as Potent Antioxidant Agents

**Figure 5:**
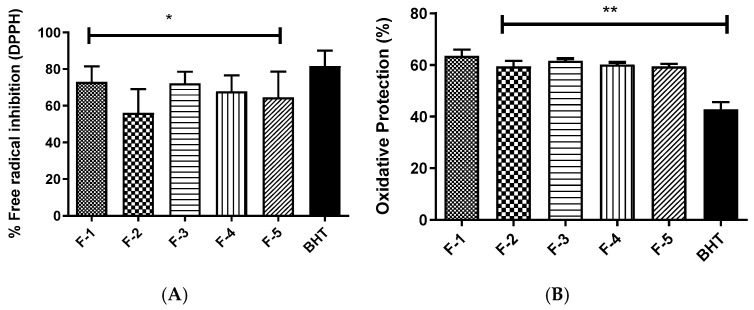
Antioxidant activity of flavonoids, with BHT as a positive control. (**A**) Antioxidant Activity (%DPPH): Evaluation of the DPPH radical scavenging capacity. (**B**) Oxidative Protection (%): Evaluation of the oxidation inhibition capacity by the *β*-carotene/linoleic acid system. Values are presented as mean ± standard deviation. (*) indicate statistically significant differences between the compared groups (* *p* < 0.05; ** *p* < 0.01, as indicated in the graph).

The flavonoids were evaluated for their antioxidant capacity using the DPPH radical scavenging assay and the *β*-carotene/linoleic acid system (Figure 5). In the DPPH assay, all five flavonoids exhibited significant antioxidant activity at 1 mg/mL concentrations. Among them, flavonoids **1**, **3**, and **5** stood out, presenting activity percentages close to that of the positive control (BHT). As detailed in Table 2, through the calculation of IC_50_, flavonoid **1** confirmed its prominence by recording the lowest IC_50_ value (0.22 ± 0.94 mg/mL), demonstrating the highest free radical scavenging capacity among all tested flavonoids and surpassing the BHT control (IC_50_ = 0.60 ± 0.09 mg/mL).

Regarding the *β*-carotene/linoleic acid system, the flavonoids also demonstrated antioxidant capacity at a concentration of 1 mg/mL. Flavonoids **1** and **2** were particularly effective, achieving the highest percentages of oxidative protection and outperforming the positive control, BHT. Under the evaluated experimental conditions, the flavonoids could significantly delay the oxidation of linoleic acid and stabilize the DPPH radical, highlighting their potent antioxidant properties.

### 2.9. Flavonoids Are Capable of Modulating Acetylcholinesterase Activity at Low Concentrations

The findings showed that the flavonoids displayed no significant inhibitory activity at the highest tested concentration (1 mg/mL). Nevertheless, they could inhibit acetylcholinesterase at lower concentrations (0.50 and 0.25 mg/mL).

**Figure 6:**
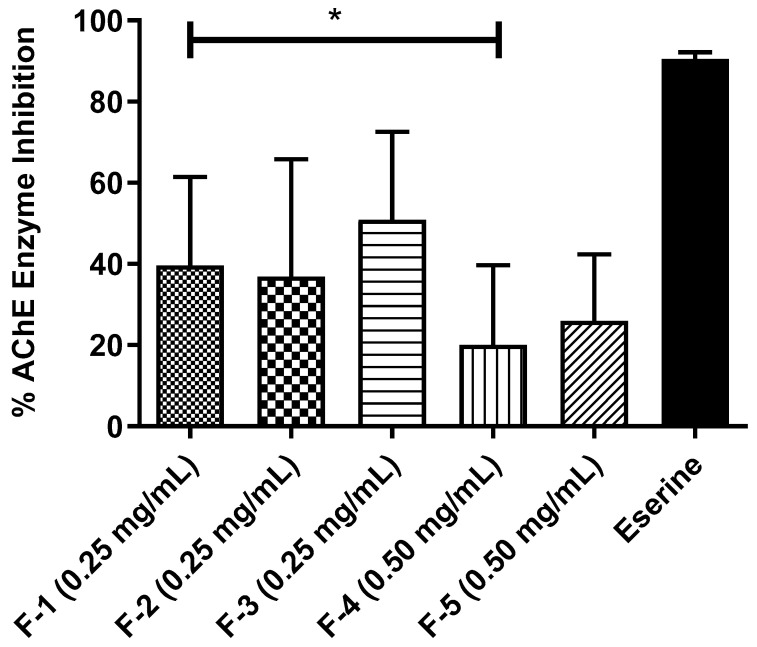
Results are expressed as mean ± standard deviation (*n* = 3). (*) indicates statistically significant difference (*p* < 0.05).

Eserine, the standard, showed the most significant inhibitory activity, reaching about 100% AChE inhibition. Flavonoids **1**, **2**, and **3** showed inhibitory potential at 0.25 mg/mL concentrations, with inhibition percentages ranging from approximately 40% to 48%. In contrast, flavonoids **4** and **5** displayed inhibition from a 0.50 mg/mL concentration, indicating lower inhibitory effectiveness with percentages between 30% and 35% (Figure 6).

## 3. Discussion

This study investigated the biological potential of flavonoids isolated from the leaves of *Erythroxylum rimosum*. The results obtained revealed a spectrum of multifaceted activities, the properties of which are intrinsically linked to their chemical structure.

The absence of cytotoxicity in mammalian cells was a highly relevant finding for most of the flavonoids, especially the quercetin glycosides (**3**, **4**, and **5**, CC_50_ > 100 μM), which corroborates previous findings demonstrating the low toxicity of *O*-glycosidic derivatives of quercetin in non-tumor cell lines [30]. The notable difference in cytotoxicity observed for flavonoid **2** (a kaempferol derivative) compared to the quercetin derivatives suggests that the absence of a hydroxyl group at the 3′ position of the B-ring may have contributed to a more pronounced cytotoxicity profile, possibly by altering its polarity and cellular interaction [31,32]. Glycosylation, in turn, tends to increase the solubility and stability of flavonoids, protecting reactive hydroxyl groups and minimizing cytotoxic impact [33]. The variability in CC_50_ values among structurally similar flavonoids (such as **2** and **3**, which have the same sugar but different aglycone backbones) reinforces that electronic rearrangements and radical substitutions in the flavonoid structure can lead to distinct biological responses, such that flavonoids with very similar structures may not generate identical biological responses [34].

Their safety profile largely supports the promising antileishmanial activity of these flavonoids. The growing resistance to conventional treatments for leishmaniasis underscores the urgency of discovering new antileishmanial agents. In this context, the promising in vitro results of flavonoids **4** and **5** against *L. braziliensis* promastigotes are amplified by the high Selectivity Index (SI) of these compounds (9.6 and 9.8, respectively), which is significantly superior to that of amphotericin B (SI = 1.4). This indicates a reduced toxicity towards mammalian cells (J774 macrophages) and greater specificity against the parasite. This favorable selectivity profile is a significant advantage in developing new therapeutic strategies and could mitigate the adverse effects associated with current drugs.

The interference capacity of flavonoids **4** and **5** was evidenced by the reduction in intracellular *L. braziliensis* infection in J774 macrophages, confirming their potential as antileishmanial agents. The observed reduction in both the percentage of infected macrophages and the number of amastigotes per field indicates that these compounds not only limit invasion but also restrict the proliferation or intracellular survival of the remaining parasites.

Flavonoid **4** showed superior performance to flavonoid **5** in both analyses, promoting a 43% reduction in the number of infected macrophages and a 40% reduction in the amastigote count, compared to the 36% and 27% reductions observed for flavonoid **5**, respectively. Despite the structural similarity between the compounds, this difference in efficacy suggests that specific structural modifications in flavonoid **4** may favor greater affinity for parasitic targets, better cellular penetration, or more efficient action on the intracellular mechanisms involved in infections.

The observed efficacy corroborates other studies investigating flavonoids as antileishmanial agents. For example, Gervanozi et al. [35] demonstrated that the flavonoid 2′-Hydroxyflavanone (2HF) significantly reduced the intracellular infection index and the number of *L. amazonensis* amastigotes in murine macrophages without exhibiting toxicity to host cells. Additionally, 2HF proved effective in a murine model of cutaneous leishmaniasis, decreasing lesion size and parasitic load in vivo without signs of systemic toxicity.

These collective results reinforce the effective interference of flavonoids with the intracellular survival of *Leishmania* in macrophages, consolidating the potential of this class of compounds as a promising source for the development of new therapeutic agents against leishmaniasis.

The morphological alterations observed by scanning electron microscopy in treated promastigotes, such as loss of membrane integrity and flagellum shortening, suggest that the flavonoids act directly on the parasite’s structure and viability. The presence of the sugar in the flavonoid structure may increase stability and bioavailability and amplify the inhibitory effect, with the fundamental backbone, binding sites, and type of sugar being crucial for the pharmacological outcome [36]. In silico studies indicate that substitution at the C-3 position of the flavonoid may be more effective for increasing the aglycone portion’s bioavailability than substitution at the C-7 position [37].

The activity of flavonoids against *Leishmania* arginases is a field of interest, as demonstrated by the inhibition of *L. amazonensis* arginase (LaARG) by isoquercetin [34]. The high sequential and structural conservation among LaARG, LbARG, and LmARG justifies using in silico models with LmARG (PDB = 4IU1) to infer interactions with LbARG. Molecular docking studies revealed that the quercetin core of flavonoids **4** and **5** interacts similarly with the catalytic site of LmARG, forming hydrogen bonds with Ser150 and Asn152, as well as hydrophobic interactions with Thr257 - residues also involved in the binding of the inhibitor nor-NOHA [29]. The ability of flavonoid **4** to form an additional hydrogen bond with Asp194 through its catechol moiety is an important finding, consistent with the interaction observed in LaARG [38]. The chiral differences between glucose (in flavonoid **4**) and galactose (in flavonoid **5**) resulted in distinct interaction profiles with the amino acids of the catalytic site (e.g., Ala192, Asn143, and Ser150 for glucose; Asp141 and Thr257 for galactose), highlighting the subtle yet significant role of the sugar type in binding selectivity. The high conservation of the binding site amino acids between LmARG and LbARG confers robustness to the extrapolation of these in silico results.

The antioxidant capacity of flavonoids is a focus of pharmaceutical research, as it is believed to be a key mechanism for their activity against various diseases. Our results confirm the high antioxidant power of these compounds. The efficacy of proton and electron donation is intrinsically linked to the number and position of hydroxyl groups on the molecule [39]. The superiority of flavonoid **1** (quercetin) as a free radical scavenger is attributed to its unique chemical structure, particularly the combination of the C-4 carbonyl group with the catechol moiety and the hydroxyl group at C-3, which allows for electron delocalization and radical stabilization [40,41].

The variation in antioxidant efficacy between flavonoids glycosylated with six-carbon sugars (compounds **4** and **5**) is noteworthy. The chiral difference at the 4-carbon between glucose and galactose, which impacts the three-dimensional arrangement of the substituents, may explain this discrepancy in activity. Similarly, the comparison between flavonoids with a pentose but different aglycones (flavonoid **3** and **2**) underscores that both the quantity and position of hydroxyl groups on the B-ring (especially the 3′,4′-catechol structure) are fundamental to the mechanisms of antioxidant activity [42]. The variability observed among the studied flavonoids highlights flavonoid **1** as the most promising.

The investigation of the antioxidant capacity of flavonoids is particularly relevant as studies indicate that oxidative stress plays a central role in the pathogenesis of various diseases, such as neurodegenerative diseases, in which the enzyme acetylcholinesterase (AChE) plays a crucial role. Compounds with antioxidant capacity can modulate the activity of this enzyme, contributing to neuroprotection and the development of new therapeutic approaches. Acetylcholinesterase (AChE) regulates acetylcholine levels in synapses and neuromuscular junctions, terminating nerve impulses. In Alzheimer’s disease, the decrease in acetylcholine is one of the main factors associated with cognitive decline. Therefore, inhibition of AChE is a key therapeutic approach for treating this condition [43].

Although the flavonoids did not exhibit significant inhibition of AChE at the highest concentration tested (1 mg/mL), they could inhibit it at lower concentrations. This behavior may be attributed to the biphasic effect of quercetin, where high doses can promote a pro-oxidant effect that could, theoretically, impair enzymatic activity [44]. The results suggest flavonoids **1**, **2**, and **3** have greater AChE inhibitory potential at reduced concentrations.

The efficacy of flavonoids in preventing neurodegenerative disorders is associated with reduced beta-amyloid (Aβ) protein toxicity and decreased oxidative stress [42,43,44,45]. The presence of a hydroxyl group at the C-3 position of the flavonoid (as in quercetin, flavonoid **1**) is relevant for metal chelation, antioxidant action, and prevention of Aβ aggregation [46]. Furthermore, hydroxyl groups on the A-ring and the C-2/C-3 double bond favor interaction with AChE, enhancing inhibition [47]. Therefore, considering the structures of the flavonoids, it is observed that flavonoids **2** and **3**, which have a pentose-type sugar in their composition, showed higher percentages of enzymatic inhibition, suggesting that this five-carbon sugar unit may have contributed to the increased inhibitory activity.

## 4. Materials and Methods

### 4.1. Obtaining Flavonoids

The flavonoids used in this study were isolated at the Natural Products Research Laboratory (LPPN) of the Faculty of Pharmacy of the Federal University of Bahia (UFBA). The chemical characterization of the flavonoids was previously described by Ribeiro et al. [28]. These flavonoids were isolated from the ethyl acetate fraction obtained from the crude methanolic extract by liquid–liquid partitioning. This fraction was subjected to silica gel column chromatography, yielding major subfractions. Further purification was carried out using Sephadex LH-20 (Supleco® Sigma-Aldrich, San Luis, MO, USA) cromatography and recrystallization. The identification and structural elucidation of the compounds were confirmed by one- and two-dimensional NMR analyses.

### 4.2. Cell Culture

J774 murine macrophage-like cells were cultured in Dulbecco’s Modified Eagle’s Medium (DMEM; Gibco, Thermo Fisher Scientific, Waltham, MA, USA), supplemented with 10% fetal bovine serum (FBS; Gibco, Thermo Fisher Scientific, Waltham, MA, USA) and 50 μg/mL of gentamicin (Life, Carlsbad, CA, USA). The cells were maintained at 37 °C in a 5% CO_2_ humidified atmosphere. Cell maintenance and all experiments were performed under aseptic conditions in a laminar flow hood. The cells were subcultured every 3–4 days and detached from culture flasks using a 1× trypsin-EDTA solution. Cell counting and viability assessment were performed using the trypan blue exclusion method. For the viability experiments, cells were seeded in 96-well plates and incubated for 24 h to allow for adherence and stabilization before treatment initiation.

### 4.3. Parasite Cultivation

The promastigote forms of *L. braziliensis* (MHOM/BR88/BA-3456) were cultured in Schneider medium (Sigma-Aldrich, San Luis, MO, USA) containing 10% fetal bovine serum (FBS, Gibco, Thermo Fisher Scientific, Waltham, MA, USA), 50 μg/mL gentamicin (Life, Carlsbad, CA, USA), pH 7.2 and incubated at 26 °C. The parasites were counted in the Neubauer chamber until they reached the stationary growth phase, and new passages were performed.

### 4.4. Evaluation of Cytotoxic Activity in Macrophages In Vitro

J774 murine macrophages were seeded in 96-well plates at a density of 2 × 10^4^ cells/well in Dulbecco’s Modified Eagle’s Medium (DMEM). The flavonoids were tested in six concentrations, in triplicate, and incubated with the cells for 72 h. After this period, 20 µL of Alamar Blue™ (ACS Cientifica, São Paulo, SP, Brazil) was added to each well, followed by an additional 6 h incubation. Cell viability was measured spectrophotometrically using a microplate reader (SpectraMax 190, Molecular Devices, Sunnyvale, CA, USA) at 570 and 600 nm wavelengths. The results were expressed as the 50% cytotoxic concentration (CC_50_), defined as the concentration that reduces cell viability by 50%. Gentian violet (Synth, São Paulo, SP, Brazil) was used as the positive control for cytotoxicity.

### 4.5. Evaluation of the Antileishmanial Activity of Flavonoids in Axenic Culture of L. braziliensis

*Leishmania braziliensis* promastigotes (1 × 10^6^ parasites/well) were cultured in 96-well plates containing Schneider’s medium supplemented with 10% fetal bovine serum (FBS) and gentamicin (50 µg/mL). The cultures were treated with different concentrations of the flavonoids (ranging from 100 to 6.25 µM). After a 72 h incubation at 26 °C, 20 µL of Alamar Blue™ was added to each well, followed by a further 2 h incubation. The viability readout was performed using a microplate reader (SpectraMax 190, Molecular Devices, Sunnyvale, CA, USA) at 570 and 600 nm wavelengths. The activity of the flavonoids was expressed as the percentage of inhibition of parasite viability compared to the negative control.

### 4.6. Evaluation of Antileishmanial Activity in Macrophages Infected with L. braziliensis

J774 murine macrophages (5 × 10^5^ cells/well) were seeded into 24-well plates containing sterile 13 mm round coverslips and incubated for 24 h (overnight). Stationary-phase *L. braziliensis* promastigotes were then added at a 10:1 parasite-to-macrophage ratio. After a 4 h infection period, the wells were treated in duplicate with flavonoids **4** and **5** at concentrations ranging from 2.5 to 20 µM and with the reference drug amphotericin B (3 µM). The negative control consisted of RPMI medium supplemented with 10% FBS, without the addition of flavonoids. After 24 h of incubation, the cells were fixed with methanol and stained with Giemsa (Dinâmica, Química Contemporânea Ltd., São Paulo, SP, Brazil). The infection was assessed by counting 100 cells per well to determine the percentage of infected macrophages and the mean number of amastigotes per macrophage. Micrographs were obtained using a camera attached to a light microscope (Leica ICC50W, Wetzlar, Germany) and analyzed with the Leica Microsystems AirLab V. 2.0. software.

### 4.7. Ultrastructural Analysis by Scanning Electron Microscopy

*L. braziliensis* promastigotes (5 × 10^7^) were incubated in microcentrifuge tubes containing 1 mL of Schneider’s medium (Sigma-Aldrich) and treated with flavonoids **4** and **5** at concentrations of 5 µM and 10 µM, as well as with the reference drug amphotericin B (3 µM), for 24 h at 26 °C. After treatment, the samples were fixed and processed for scanning electron microscopy analysis. Initially, the parasites were fixed in a 2% glutaraldehyde solution prepared in 0.1 M sodium cacodylate buffer for 2 h at room temperature. Subsequently, the samples were post-fixed in a solution containing 1% osmium tetroxide in 0.1 M cacodylate buffer for 1 h at room temperature. After the fixation steps, the parasites were adhered to glass coverslips treated with 0.01% poly-L-lysine, dehydrated in an ascending ethanol series (30% to 100%), and subjected to critical point drying with liquid CO_2_ replacing the ethanol. Afterwards, the samples were sputter-coated with gold and analyzed using a JEOL JSM-6390LV scanning electron microscope (JEOL, Chiyoda-ku, Tokyo, Japan).

### 4.8. In Silico Study of Molecular Docking

The GOLD 5.3.0 program [48] using the ChemPLP function, was employed to perform the docking of flavonoids **4** and **5** into the catalytic site of the *L. mexicana* arginase (LmARG) structure, obtained from the Protein Data Bank (PDB ID: 4IU1) [29]. Prior to the docking calculations, the compound structures were drawn using the Marvin Sketch 16.9.5 program (ChemAxon Ltd., Budapeste, Hungary) [49] through which the most reliable tautomers of the compounds were also selected, considering the protonation profile at pH = 7.0. Subsequently, the CONCORD module of SYBYL^®^-X 2.0 [50] was used to generate the 3D structures of the inhibitors. The optimization of the 3D inhibitor structures was carried out using the Conjugate Gradient (CG) method with a convergence criterion of 0.001 kcal mol^−1^, employing the Tripos force field (ε = 80.4) and setting a maximum of 50,000 interactions [51]. Furthermore, Gasteiger-Hückel charges [47] were assigned to the compounds in the SYBYL^®^-X 2.0 platform [50]. Also, prior to the calculations, the GOLD 5.3.0 program [45] was evaluated for its ability to reproduce the pose of the competitive inhibitor nor-NOHA, co-crystallized within the binding site of the *L. mexicana* arginase structure. The results were analyzed using the Pymol [52] and PoseView [53] programs.

### 4.9. DPPH Radical Sweeping Method

A stock solution of the tested flavonoids was prepared in methanol at 5.0 mg/mL. From this solution, serial dilutions (1:2) were performed, resulting in different concentrations ranging from 2.5 mg/mL to 0.03906 mg/mL. The assay was conducted in triplicate in a 96-well plate by adding 50 µL of each flavonoid solution and 200 µL of a (DPPH) 2,2-diphenyl-1-picrylhydrazyl (Sigma-Aldrich™, St. Louis, MO, USA) solution. Butylated hydroxytoluene (BHT, Êxodo Científica™, Sumaré, SP, Brazil) was used as the positive control and methanol as the negative control. The plate was incubated for 40 min in the dark, and the reduction in the DPPH radical was monitored by measuring the absorbance at 600 nm. The percentage of antioxidant activity was calculated using the following formula:

% Antioxidant = [(DPPH_Abs_ − Sample_Abs_)/(DPPH_Abs_)] × 100 (1)

The IC50 value, defined as the antioxidant concentration required to reduce the initial DPPH concentration by 50%, was calculated using the equation of the straight line from the inhibition curve in relation to the concentration of the compounds.

### 4.10. Method for Inhibiting the Oxidation of β-Carotene/Linoleic Acid

The flavonoids were diluted in methanol to obtain a stock concentration of 1.0 mg/mL. To prepare the emulsion, 0.5 mg of *β*-carotene (Sigma-Aldrich™, St. Louis, MO, USA) was dissolved in 1 mL of chloroform and transferred to a 200 mL round-bottom flask, to which 200 μL of Tween 80 (Synth, Diadema, SP, Brazil) and 25 μL of linoleic acid were added. After the evaporation of the chloroform, 50 mL of water was added to form the emulsion.

In a 96-well plate, 160 μL of the β-carotene/linoleic acid emulsion and 40 μL of each tested flavonoid were added. For the positive control, 40 μL of BHT was used, and the negative control consisted of substituting the sample with 40 μL of methanol. All assays were performed in triplicate.

The discoloration kinetics of the emulsion were monitored by spectrophotometry at 470 nm (microplate reader, SpectraMax 190, Molecular Devices, Sunnyvale, CA, USA) before incubation (T_0_) and at intervals of 30, 60, 90, and 120 min at 50 °C. The antioxidant activity was expressed as a percentage of inhibition, calculated by the following equation:

% Inhibition = (Abic − Abfc) − (Abiam − Abfam) × 100(2)

where Abic and Abfc are the initial and final absorbances of the control, and Abiam and Abfam are the initial and final absorbances of the sample, respectively.

### 4.11. Acetylcholinesterase Inhibition Method

The acetylcholinesterase inhibition assay was performed in microplates with the following reaction mixture: 140 μL of phosphate buffer (pH 8.0), 10 μL of acetylcholinesterase enzyme (0.5 U/mL), 20 μL of 5,5′-dithiobis (2-nitrobenzoic acid) (DTNB, 10 mM) (Thermo Scientific™, Waltham, MA, USA), and 20 μL of the sample (flavonoid). The initial absorbance was read at 405 nm. The reaction was initiated by adding 20 μL of acetylcholine iodide (Sigma-Aldrich™, St. Louis, MO, USA). The eserine (physostigmine) standard was used as the positive control, and methanol as the negative control. The microplate was protected from light, and the reaction was monitored by measuring the absorbance at 405 nm every minute for 10 min.

The percentage of inhibition was calculated using the following formula:

%I = (Abc − Abam)/Abc × 100(3)

where Abc corresponds to the absorbance of the control and Abam to the absorbance of the sample.

### 4.12. Statistical Analysis

The analyses conducted in this study were performed in triplicate, and the results are expressed as mean ± standard deviation (*n* = 3) to ensure greater statistical reliability and data reproducibility. To assess potential differences between the experimental groups, one-way analysis of variance (ANOVA) was applied, a procedure widely used in comparative studies involving multiple samples. When ANOVA identified a significant difference, Tukey’s multiple comparison test was used, adopting a 95% confidence level, to determine which groups presented statistically significant differences. Any difference with a probability of error of less than 5% (*p* < 0.05) was considered statistically significant, i.e., with a low chance that the observed difference occurred by chance. For better interpretation of the results, the significance values were organized into categories according to the level of statistical evidence: **** *p* < 0.0001 (extremely significant), *** 0.0001 ≤ *p* < 0.001 (very significant), ** 0.001 ≤ *p* < 0.01 (significant), * 0.01 ≤ *p* < 0.05 (slightly significant) and ns *p* ≥ 0.05 (not significant), indicating no statistical difference between the compared groups. All statistical analyses were processed using GraphPad Prism™ software version 10.1 (GraphPad Software, Inc., San Diego, CA, USA), a recognized tool for the statistical treatment of biological data and the graphical presentation of results.

## 5. Conclusions

The flavonoids evaluated demonstrated selective anti-Leishmania activity and were not toxic to mammalian cells. Specifically, the glycosylated derivatives Quercetin-3-*O*-*β*-glucopyranoside (**4**) and Quercetin-3-*O*-*β*-galactopyranoside (**5**) in *β*-hexose configuration were able to significantly reduce both the infection rate of J774 macrophages and the number of intracellular amastigotes. The flavonoid Quercetin-3-*O*-*β*-glucopyranoside was more effective than Quercetin-3-*O*-*β*-galactopyranoside, suggesting that its specific structural modifications optimize its penetration into the cell and/or its interaction with molecular targets. Treatment with flavonoids caused severe damage to the morphology of the parasites, including membrane bulging, drastic shortening of the flagellum, and loss of the fusiform shape (becoming spherical), compromising their structural integrity and viability. Molecular docking results showed that the type of sugar (glucose vs. galactose) and extra hydrogen bonds (such as that of flavonoid **4** with Asp194) are factors that differentiate and optimize the binding of each compound. There was a clear structure–activity relationship in antioxidant activity; the aglycone quercetin (**1**), because of its free hydroxyl groups, demonstrated a much greater free radical scavenging capacity than its glycosylated derivatives.

Therefore, in the evaluation of acetylcholinesterase (AChE) inhibition, the pentose-containing flavonoids Kaempferol-3-*O*-*α*-arabinofuranoside (**2**) and Quercetin-3-*O*-*α*-arabinofuranoside (**3**) exhibited mild-to-moderate activity, indicating that the nature of the sugar also modulates this function. Overall, flavonoids emerge as a class of compounds with remarkable pharmacological versatility, capable of exerting a broad spectrum of biological actions. It is clear that their bioactive potential is not uniform, but rather directly modulated by their structural characteristics, such as their basic skeleton and the type of substitution of the functional groups, whose activity can be finely tuned by chemical modifications to optimize a desired therapeutic effect.

## Figures and Tables

**Table 1:** Cytotoxicity profile, antileishmanial activity and selectivity index of flavonoids isolated from *Erythroxylum rimosum*.

Flavonoids	J774 Macrophages CC_50_ Mφ (μM)	*L. braziliensis* Promastigotes IC_50_ ± SD (μM)	Selectivity Index (CC_50_/IC_50_)
**1**	-	-	-
**2**	29.40 ± 0.30	>100	-
**3**	>100	54.70 ± 0.20	1.8
**4**	>100	10.34 ± 0.70	9.6
**5**	>100	10.14 ± 0.50	9.8
Amphotericin B	3.30 ± 0.50	2.28 ± 0.20	1.4
Gentian Violet	0.30 ± 0.20	ND	ND

**Table 2:** Determination of antioxidant activity by the DPPH free radical scavenging method of flavonoids under 50% inhibition.

Flavonoids	IC_50_ mg/mL
**1**	0.22 ± 0.94
**2**	2.46 ± 1.44
**3**	0.61 ± 0.07
**4**	0.42 ± 0.09
**5**	1.37 ± 0.15
BHT	0.60 ± 0.09

## Data Availability

The raw data supporting the conclusions of this article will be made available by the authors on request.

## Supplementary Materials

The following supporting information can be downloaded at: https://www.mdpi.com/article/10.3390/molecules31101669/s1. Table S1: Global sequence alignment between amino acid residues of the arginase enzyme from *Leishmania mericana* (LmArg), *L. braziliensis* (LbArg) and *L. amazonensis* (LaArg). Global sequence identity percentage: LbArg × LmArg = 84.8%; LbArg × LaArg = 86.9%; LmArg × LaArg = 95.74%. Local sequence identity percentage of the catalytic region: LbArg × LmArg = 88.2%; LbArg × LaArg = 88.2%; LmArg × LaArg = 100.0%. Residues that compose the catalytic site region are highlighted. (“*” = Identical, “:” = Similar, “.” = semi-conserved, “ ” = Not conserved, “-” = absent). Figure S1: Superposition between the pose of the nor-noha inhibitor obtained from the molecular docking calculation with the GOLD 5.3.0 program (PLP function) (represented in yellow stick) in the catalytic site of *L. mexicana* arginase (LmARG; PDB ID = 4IU1) and the atomic coordinates of the same inhibitor from the crystallographic complex (represented in red stick). RMSD = 1.55Å. LmARG represented in cartoon.
