# Supplementary material for: Structure–Activity Relationship Analysis of Flavonoids Isolated from the Leaves of Erythroxylum rimosum O. E. Schulz
Source: Molecules. 2026 May 15;31(10):1669. doi: 10.3390/molecules31101669 (PMC13209892; doi:10.3390/molecules31101669)
Supplement: Supplementary file 1 [file molecules-31-01669-s001.zip › molecules-3908529-supplementary.pdf]

Structure–Activity Relationship Analysis of Flavonoids Isolated from the Leaves of *Erythroxylum rimosum* O. E. Schulz

Ivana P. dos Santos [1], Maria Eduarda V. Costa [2], Geovana A. de Oliveira [2], Francisnaira S. Santos [1], Débora O. S. Vitória [1], Robson Almeida Silva [3], Janaina M. C. do Vale [3], Gildeon S. Marques [3], Ademir Evangelista do Vale [3], Milena Botelho Pereira Soares [4,5], Taise de A. Araújo [1], André Braga Teles [1], Elisalva Teixeira Guimarães [1,4] and Erika Maria de Oliveira Ribeiro [1,*]

[1] Departamento de Ciências da Vida, Universidade Estadual da Bahia, Salvador 41150-000, Brazil; ivana.pfarma@gmail.com (I.P.d.S.); santosnaira89@gmail.com (F.S.S.); debivic02@gmail.com (D.O.S.V.); taiandradearaujo@gmail.com (T.d.A.A.); alteles@uneb.br (A.B.T.); etguimaraes@uneb.br (E.T.G.)

[2] Escola Bahiana de Medicina e Saúde Pública, Salvador 41150-000, Brazil; mecosta2510@gmail.com (M.E.V.C.); geovana.alvesdeoli@gmail.com (G.A.d.O.)

[3] Faculdade de Farmácia, Universidade Federal da Bahia, Salvador 40170-115, Brazil; robsonalmeida74@gmail.com (R.A.S.); janaynadovale@gmail.com (J.M.C.d.V.); gil_silv@hotmail.com (G.S.M.); advale@gmail.com (A.E.d.V.)

[4] Instituto Gonçalo Moniz, Salvador 40296-710, Brazil; milena.soares@fiocruz.br

[5] Instituto SENAI de Inovação em Sistemas Avançados em Saúde (CIMATEC ISI SAS), Salvador 41650-010, Brazil

* Correspondence: eribeiro@uneb.br

SUPPLEMENTARY MATERIAL

Table S1: Global sequence alignment between amino acid residues of the arginase enzyme from Leishmania mexicana (LmArg), L. braziliensis (LbArg) and L. amazonensis (LaArg). Global sequence identity percentage: LbArg x LmArg = 84.8%; LbArg x LaArg = 86.9% ; LmArg x LaArg = 95.74%. Local sequence identity percentage of the catalytic region: LbArg x LmArg = 88.2%; LbArg x LaArg = 88.2% ; LmArg x LaArg = 100.0%. Residues that compose the catalytic site region are highlighted. ("*" = Identical, ":" = Similar, "." = semi-conserved, " " = Not conserved, "-" = absent).

```
LbArg    MEHHLQKYKFYKEKNMSIVLAPFSGGQPLSGVELGPDYLLKQGLQQDMEKLGWNTTLERV    60
LmArg    MRGSHHHHHHGMAKKMSIVLAPFSGGQPHSGVELGPDYLLKQGLQQDMEKLGWDTRLERV    60
LaArg    -MEHVQQYKFYKEKKMSIVLAPFSGGQPHSGVELGPDYLLKQGLQQDMEKLGWDTRLERV    59
              ::::.   *:************* ************************:* ****

LbArg    FDGKIVEARKANEKNDLIGHIKRPKLTSECTEKIYNSVRKVAEQGRFPLTVGGDHSIAVG    120
LmArg    FDGKVVEARKASDNGDRIGRVKRPRLTAECTEKIYKCVRRVAEQGRFPLTIGGDHSIALG    120
LaArg    FDGKVVEARKASDNGDRIGRVKRPRLTAECTEKIYKCVRRVAEQGRFPLTIGGDHSIALG    119
         ****:******.::.* **::***:**:*******:.**:**********:*******:*

LbArg    TVAGVLSVYPDTGVIWVDAHADINTMSGTVSGNLHGCPLSILLGLDRENIPECFSWVPQL    180
LmArg    TVAGVLSVHPDAGVIWVDAHADINTMSGTVSGNLHGCPLSILLGLDRENIPECFSWVPQV    180
LaArg    TVAGVLSVHPDAGVIWVDAHADINTMSGTVSGNLHGCPLSILLGLDRENIPECFSWVPQV    179
         ********:**:***********************************************:

LbArg    LKPHKIAYIGLRDVEEAEKKILHDLNIAAFSMHHVDRYGIDKVVRMAIDAVSPKGTEPVM    240
LmArg    LKPNKIAYIGLRAVDDEEKKILHDLNIAAFSMHHVDRYGIDKVVSMAIEAVSPKGTEPVM    240
LaArg    LKPNKIAYIGLRAVDDEEKKILHDLNIAAFSMHHVDRYGIDKVVSMAIEAVSPKGTEPVM    239
         ***:******** *:: *************************** ***:***********

LbArg    VSYDVDTIDPLYVPATGTPVRGGLSLREGLFLCERIAECGRLVALDVVECNPLLAATEAH    300
LmArg    VSYDVDTIDPLYVPATGTPVRGGLSFREALFLCERIAECGRLVALDVVECNPLLAATESH    300
```

```
LaArg      VSYDVDTIDPLYVPATGTPVRGGLSFREALFLCERIAECGRLVALDVVECNPLLAATESH    299
           ************************:**.****************************:*

LbArg      VKDTISFGCAIARCMMGETLLYTPRKKAKL      330
LmArg      VNDTISVGCAIARCMMGETLLYTPHTSSKL      330
LaArg      VNDTISDGRAIARCMMGETLLYTPHTSSKL      329
           *:**** * ***************:..:**
```

Figure S1: Superposition between the pose of the nor-noha inhibitor obtained from the molecular docking calculation with the GOLD 5.3.0 program (PLP function) (represented in yellow stick) in the catalytic site of L. mexicana arginase (LmARG; PDB ID = 4IU1) and the atomic coordinates of the same inhibitor from the crystallographic complex (represented in red stick). RMSD = 1.55Å. LmARG represented in cartoon.

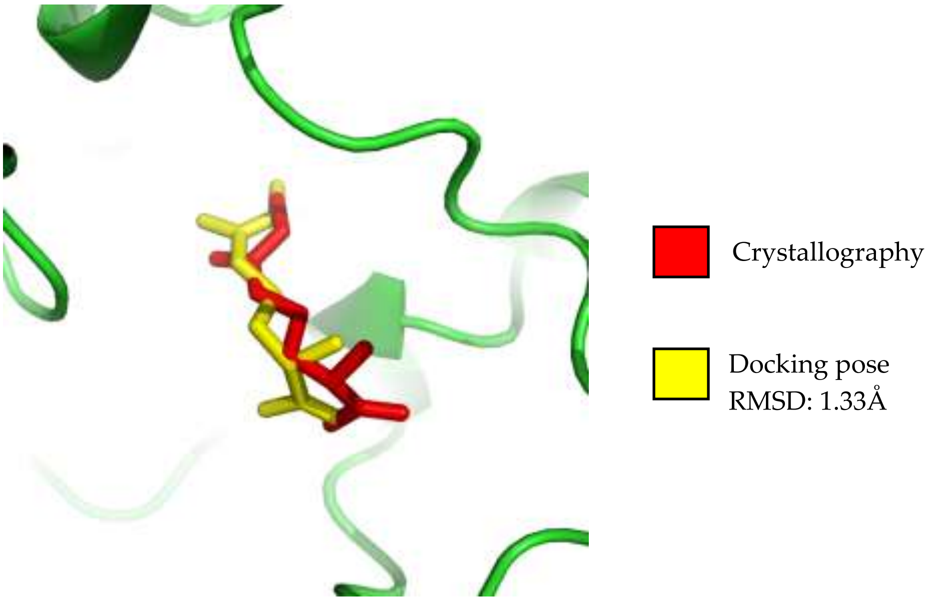

Author Contributions: Investigation, methodology, data acquisition, writing of the original draft: IVANA P. DOS SANTOS and ROBSON ALMEIDA SILVA; Investigation, methodology, data acquisition: FRANCISNAIRA S. SANTOS, JANAINA M. C. DO VALE and GILDEON S. MARQUES; Methodology, data acquisition: MARIA EDUARDA V. COSTA, GEOVANA A. DE OLIVEIRA, DÉBORA O. S. VITÓRIA and TAISE DE A. ARAÚJO; Resources, funding acquisition: ADEMIR E. DO VALE and MILENA B. P. SOARES; Software, validation: ANDRÉ BRAGA TELES; Conceptualization, methodology, formal analysis, review, validation and supervision: ELISALVA T. GUIMARÃES and ERIKA M. DE O. RIBEIRO.
